# Prey exploitation and dispersal strategies vary among natural populations of a predatory mite

**DOI:** 10.1002/ece3.4446

**Published:** 2018-10-13

**Authors:** Alexandra M. Revynthi, Martijn Egas, Arne Janssen, Maurice W. Sabelis

**Affiliations:** ^1^ Institute of Biodiversity and Ecosystem Dynamics University of Amsterdam Amsterdam The Netherlands

**Keywords:** behavioral syndrome, Killer strategy, metapopulations, Milker strategy, *Phytoseiulus persimilis*

## Abstract

When predators commonly overexploit local prey populations, dispersal drives the dynamics in local patches, which together form a metapopulation. Two extremes in a continuum of dispersal strategies are distinguished: the “Killer” strategy, where predators only start dispersing when all prey are eliminated, and the “Milker” strategy, in which predator dispersal occurs irrespective of prey availability. Theory shows that the Milker strategy is not evolutionarily stable if local populations are well connected by dispersal. Using strains of the predatory mite *Phytoseiulus persimilis*, collected from 11 native populations from coastal areas in Turkey and Sicily, we investigated whether these two strategies occur in nature. In small wind tunnels, we measured dispersal rates and population dynamics of all populations in a system consisting of detached rose leaves, spider mites (*Tetranychus urticae*) as prey, and *P. persimilis*. We found significant variation in the exploitation and dispersal strategies among predator populations, but none of the collected strains showed the extreme Killer or Milker strategy. The results suggest that there is genetic variation for prey exploitation and dispersal strategies. Thus, different dispersal strategies in the Milker–Killer continuum may be selected for under natural conditions. This may affect the predator–prey dynamics in local populations and is likely to determine persistence of predator–prey systems at the metapopulation level.

## 1 INTRODUCTION

Dispersal is a key process in population biology, influencing the persistence, distribution, and abundance of populations as well as driving gene flow (Dingle, 1996; Dunley & Croft, 1990; Quinn, Cole, Patrick, & Sheldon, 2011). Decisions of individuals to disperse typically depend on local conditions such as the local density of conspecifics in the same patch (Otronen & Hanski, 1983), food availability (Kuussaari, Nieminen, & Hanski, 1996), interspecific interactions (Weisser, McCoy, & Boulinier, 2001), sex ratio (Colwell & Naeem, 1999; Lawrence, 1987, 1988), kin recognition and kin interactions (Hamilton & May, 1977), inbreeding avoidance (Greenwood, 1980; Pusey & Wolf, 1996), cannibalism (Pels, 2001), individual personality (Quinn et al., 2011), temporal and spatial heterogeneity (Holt & Barfield, 2001; Wiens, 2001), and patch isolation (Conradt, Roper, & Thomas, 2001).

Dispersal affects various levels of biological organization, from an individual's fitness to population dynamics and community composition (Bowler & Benton, 2005). Dispersal is particularly important when local populations are driven to extinction because of overexploitation, whereas persistence is observed at a metapopulation level due to frequent foundations of new local populations by dispersing individuals. Such dynamics occur, for example, when both predator and prey disperse at sufficient rates to balance local extinction with recolonization (Ellner et al., 2001; Huffaker, 1958; Janssen, van Gool, Lingeman, Jacas, & van de Klashorst, 1997; Taylor, 1990). When individuals disperse from their patches at different time points, this creates asynchronous fluctuations in local abundance, which are a prerequisite for the persistence of a metapopulation composed of locally unstable populations (Holyoak & Lawler, 1996).

Whereas the role of dispersal in metapopulation persistence of systems characterized by local overexploitation received much attention, the effects of dispersal on local population dynamics are less well understood (Bowler & Benton, 2009). The consequences of predator dispersal for the population dynamics of predators and prey were modeled by van Baalen and Sabelis (1995), who defined the so‐called Milker–Killer dilemma, describing under which conditions these extremes of a continuum of dispersal strategies can evolve. Predators with the Killer strategy disperse only when the prey are eliminated. Under the Milker strategy, predators will disperse irrespective of prey density, thereby decreasing predation and allowing the prey population to increase in size for a longer period. As a result, the predators’ offspring will have more food, resulting in a longer interaction period of predators and prey on the patch and consequently, a higher total number of predators produced on a patch. In contrast to the Killer strategy, the Milker strategy is a less selfish strategy (van Baalen & Sabelis, 1995), in which the predators show a more prudent exploitative behavior. The Milker strategy is not evolutionarily stable because a local population of Milkers can be invaded by Killers, which have a reproductive benefit because they exploit the prey left behind by dispersing Milkers. When there is a low probability of invasion of Milker patches by Killers, the evolution of Milkers may be favored at the metapopulation level because of the higher total number of offspring produced by a local population of Milkers than by Killers (van Baalen & Sabelis, 1995; Pels, 2001).

Depending on the dispersal rate during the predator–prey interaction, two consequences on the population dynamics of prey and predators are predicted. First, the local interaction period between a Killer predator population and its prey will be shorter than that of a Milker population. Second, local populations of Killer predators will produce less offspring over the entire local predator–prey interaction than Milker predators.

The formulation of the Milker–Killer dilemma was inspired by a study of plant‐inhabiting mites, specifically the predatory mite *Phytoseiulus persimilis* Athias‐Henriot and its prey, the phytophagous spider mite *Tetranychus urticae* Koch. This spider‐mite species occurs in local populations, which can be locally driven to extinction by their predators (Janssen & Sabelis, 1992). These local populations are connected by dispersal (Ellner et al., 2001; Janssen et al., 1997). An experimental study investigated whether both dispersal strategies occur among predator strains of *P. persimilis* (Pels & Sabelis, 1999), originally sampled from wild populations along the coast and inland on Sicily (Italy). Pels and Sabelis (1999) showed that all predator strains exterminated local prey populations, and the timing of dispersal appeared to have a genetic basis: One isofemale line derived from a coastal strain consistently showed dispersal close to or after prey elimination, whereas an isofemale line derived from an inland strain consistently dispersed long before all prey were eliminated. These behaviors were in line with Killer‐ and Milker‐like strategies, respectively.

In their study, Pels and Sabelis (1999) did not replicate measurements of dispersal behavior of the strains. Instead, they chose two strains that showed the most extreme differences in dispersal behavior and created one isofemale line from each, which they used for further experiments. Thus, dispersal strategies were characterized for only two isofemale lines and hence a thorough survey of predator dispersal behavior among natural populations is lacking. Also, quantifying the extent of variation in dispersal strategies from natural populations allows testing the predictions on the population dynamics of Killers and Milkers with their prey. We therefore returned to the Mediterranean area to collect natural populations of *P. persimilis* and measured their dispersal characteristics using local populations in a laboratory setup similarly but more accurately than described in Pels and Sabelis (1999). We aimed to quantify the extent of variation in dispersal strategies among the sampled populations by estimating dispersal rates in the presence of prey and to test the predicted consequences of dispersal for the population dynamics of predators and prey.

## 2 MATERIALS AND METHODS

### 2.1 Collection of predatory mites

*Phytoseiulus persimilis* populations were collected from fields in Turkey in 2013 and in Sicily in 2014. These locations were chosen because natural populations of this predator occur there, and there were no known cases where it had been released as a biological control agent. Upon spotting the predators in spider‐mite colonies, infested leaves with prey and predators were transferred inside plastic bottles that were closed and had an air inlet covered with mite‐proof gauze (80 μm).

**Figure 1:**
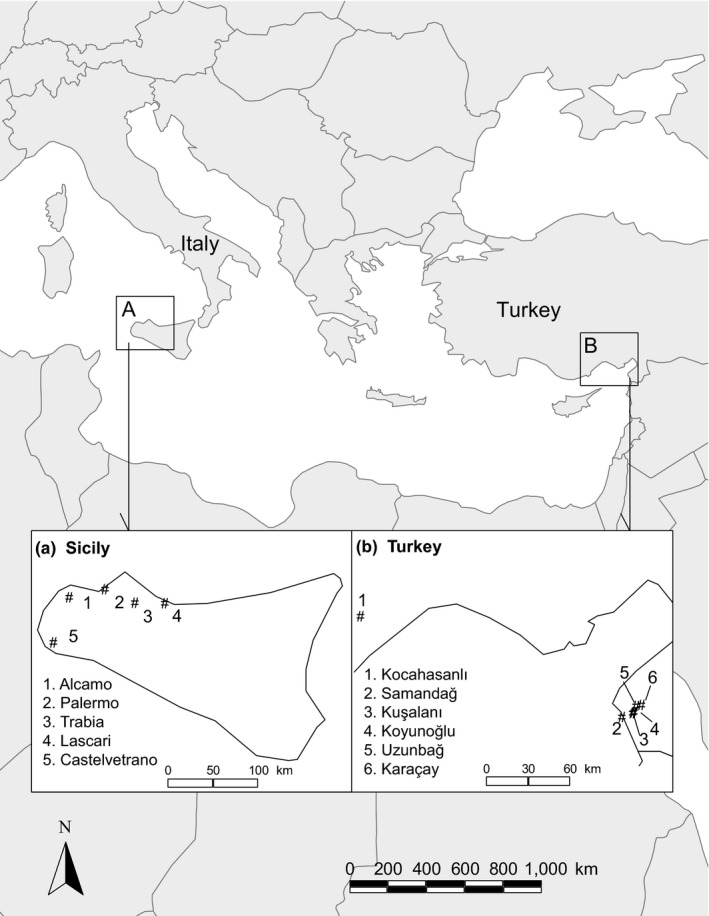
Map showing the sample sites of populations of the predatory mite *Phytoseiulus persimilis*

In Turkey, samples were collected from six different sites in the region of Hatay (36°04.950′N 035°56.728′E) and Erdemlı (36°36.267′N 034°15.926′E) (Figure 1). In Hatay, predators were collected from Samandag, Koyunoglu, Kusalani, Karacay, and Uzunbag; in Erdemli from Kocahasanli. Predatory mites were found on cucumber (*Cucumis sativus*), bean (*Phaseolus vulgaris*), and eggplant (*Solanum melongena*). In Erdemli, predators were also collected from the weed *Tribulus terrestris*. All host plants were infested with spider mites (*T. urticae*).

Predators were also collected from the western part of Sicily (38°02.573′N 012°59.747′E) at five different sites: Castelvetrano, Alcamo, Palermo, Trabia, and Lascari (Figure 1). Natural populations of *P. persimilis* occurred on spider‐mite‐infested melon (*Curcubita pepo*) and castor bean plants (*Ricinus communis*) and on spider‐mite‐infested weeds (*Convolvulus* sp).

All sampling sites in both locations where the mites were collected were along the coast. We also visited inland sites (approx. 50 km away from the coast), but did not find any predators. Predators were mostly found on plants located along the road, at small farms, and in house gardens.

### 2.2 Laboratory cultures

Rose plants (*Rosa* sp. var. Avalanche) were provided by Olij Rozen and were allowed to grow in a climate room (25°C, 70% RH, 16L: 8D) free of herbivores. Lima bean plants (*Phaseolus lunatus* L.) were grown from seeds in a climate room (25°C, 60% RH, 16L: 8D) free of herbivores. The *T. urticae* strain used to feed the predators was originally collected from cucumber plants in a commercial greenhouse in May 1994 (Pallini, Janssen, & Sabelis, 1997) and was reared on lima beans (*P. lunatus*) in a climate room (26°C, 60% RH, 16L: 8D).

The predatory mite strains were reared on spider‐mite‐infested Lima bean leaves in a walk‐in climate room at 25°C, 70% RH, and 16L:8D. We used the same closed rearing system as Pels and Sabelis (1999), consisting of a plastic float inside a plastic tray, which was filled with a 15 mm layer of water with dissolved soap. Three times per week, two bean leaflets infested with spider mites were placed on the float, which provided the predators with sufficient food. In order to allow the mites to disperse ambulatorily without drowning, the plastic float was covered with a plastic aquarium (19.5 × 13.0 × 11.5 cm) with a piece of fine‐meshed gauze hanging from the ceiling, touching the float/leaflets. For ventilation, a rectangular hole was made in the ceiling of the aquarium, covered with fine‐meshed gauze (80 μm).

### 2.3 Sequencing of COI and ITS genes

To identify the collected strains to the species level, the mitochondrial cytochrome oxidase I (COI) gene and the internal transcribed spacer (ITS) gene were sequenced. DNA was extracted from single adult females of *P. persimilis* with the Chelex maceration method (Walsh, Metzger, & Higuchi, 1991). Five mites per strain were used for the DNA extraction and were introduced individually in 0.5‐ml tubes containing 100 μl of 5% Chelex 100 (Bio‐Rad Chelex 100, Richmond, CA). The samples were incubated at 56°C with 5 μl of proteinase‐K for 60 min and were then heated for 10 min at 95°C. They were stored in the freezer at −20°C.

The mitochondrial COI region was amplified using the 5′GGTCAACAAATCATAAAGATATTGG3′ (forward) and 5′TAAACTTCAGGGTGACCAAAAAATCA3′ (reverse) primers (Jørgensen, Møbjerg, & Kristensen, 2007). The primers that were used for amplifying the nuclear ITS region were 5′AGAGGAAGTAAAAGTCGTAACAAG3′ (forward) and 5′ATATGCTTAAATTCAGGGGG3′ (reverse) (Navajas, Lagnel, Fauvel, & De, 1999). For the PCR, we used 25 μl reaction volumes containing 13.3 μl water, 2.5 μl of 10× Buffer (HT Biotechnology, Cambridge, UK), 0.5 μl Super Taq polymerase (5 U/μl), 2.5 μl dNTPs, 1.2 μl BSA, 0.5 μl of each primer, and 4 μl of DNA sample. For COI, samples were preheated at 94°C for 2 min, 35 cycles of denaturation at 94°C for 10 s, annealing at 48°C for 30 s and amplification at 72°C for 55 s, and a final extension step at 72°C for 10 min (Jørgensen et al., 2007). For ITS, samples were denatured at 94°C for 4 min, and then, PCR was carried out for 30 cycles of 1 min denaturation at 93°C, 1 min annealing at 50°C, and 1 min extension at 72°C (Navajas et al., 1999). The PCR products were visualized with UV light using a 1.5% agarose gel stained with ethidium bromide. Direct sequencing of PCR amplifications was carried out by Macrogen EZ‐seq service, using the same primers as for the PCR. The sequences were read and compared using the CLC Genomics workbench 3 (Qiagen, CLC Bio).

### 2.4 Dispersal experiments

**Figure 2:**
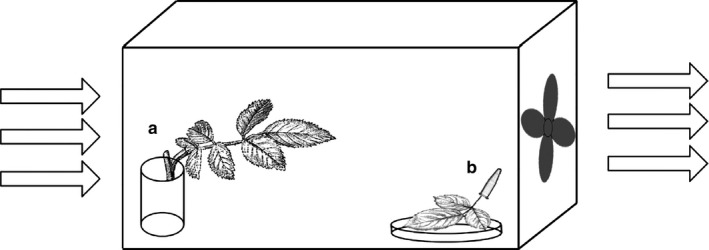
Wind tunnel, consisting of a plastic aquarium with two holes covered with mite‐proof gauze and a fan connected to one of them. (a) Experimental rose leaf, where the prey and predator were released, (b) Trap with spider‐mite‐infested rose leaf. Arrows indicate the direction of the air current

To measure dispersal, we used wind tunnels similar to those of Pels and Sabelis (1999). They consisted of a plastic aquarium (25.3 × 15.8 × 15.5 cm) with two holes on the short sides (Ø 11.5 cm each) covered with mite‐proof gauze (80 μm). The aquarium was closed with a glass lid and sealed with parafilm. A fan was placed close to one hole, causing an air flow of 0.4 m/s inside the aquarium (Figure 2).

Initially, Lima bean (*P. lunatus*) was used as a host as in Pels and Sabelis (1999). However, the leaves wilted within 24 hr after being cut. We therefore used rose leaves, which could be preserved for a long period without suffering from water stress. Rose leaves with a shoot of ca. 5 cm were cut, and the shoots were inserted in a small vial (24.5 mm Ø × 40 mm height) filled with wet floral foam (Oasis) to maintain leaf turgidity. This leaf served as the experimental leaf. A thick layer of lanolin was applied to the base of the petiole to prevent mites from escaping. Fifteen 2‐day‐old adult female spider mites were introduced on the leaf and were allowed to feed and oviposit for 2 days. Missing females were replaced daily during these 2 days. After 48 hr, one 2‐day‐old mated adult female predatory mite was introduced on the leaf, which was then placed with the vial at the upwind side in the wind tunnel. Predatory mites that dispersed aerially from the leaf using the air flow through the wind tunnel landed somewhere inside the wind tunnel. In order to capture them, we introduced a Petri dish with a young, spider‐mite‐infested rose leaf as a trap, located on the downwind side on the bottom of the wind tunnel. This trap leaf also had a ca. 3‐cm shoot, which was inserted through a hole in the lid of an Eppendorf tube (1.5 ml) filled with wet floral foam, providing it with the necessary moisture. The tube was sealed with parafilm (Figure 2). After introduction of the predator, the numbers of adult prey and all stages of predators on the experimental leaf were assessed daily, as well as the numbers of predators (all stages) on the trap leaf and elsewhere in the wind tunnel. We did not count the immature prey individuals; however, we did keep a record of their presence on the leaf. The experiment ended when there were no prey and predators of any stage left on the experimental leaf. There were five replicates, each in a separate block in time, for each strain in a climate room at 25°C, 70% RH, 16L: 8D.

Per country of origin (i.e., Sicily or Turkey), each block consisted of one replicate per strain, that is, strains from the same country were tested simultaneously, using six wind tunnels in one climate room. A line derived from mites of Koppert Biological Systems (Berkel en Rodenrijs, the Netherlands) was tested together with the Sicilian strains. This line has been maintained in our laboratory for many years and was used to contrast dispersal behavior of a laboratory population with that of the natural populations.

Specifically, we aimed at finding significant differences among strains with respect to three parameters: the dispersal rate during the predator–prey interaction, the time it took to exterminate the prey population on the experimental leaf (the so‐called interaction period), and the total numbers of predators produced on an experimental leaf. Dispersal of Milker‐like strains occurs throughout the interaction period, that is, when prey are still present. At the end of the interaction period, however, Milkers and Killers will both disperse due to lack of prey. To discriminate among more Milker‐like and Killer‐like strains, we therefore excluded the last part of the interaction period for comparison of dispersal behavior. We defined the first part of the interaction period as the time where three or more adult prey individuals were still present on the experimental leaf. Below, we refer to the dispersal taking place during the first part of the interaction period. The dispersers were removed when counting; hence, the predators found on the wind tunnel surface and the trap leaf had dispersed during the previous 24 hr. The predator stages that were observed to disperse were adult males and females, protonymphs and deutonymphs. Per time step, the dispersed predators were removed and scored, and the adult, protonymph and deutonymph predators present on the experimental leaf at the last day of dispersal during the interaction (see above) were scored as not having dispersed. A time‐to‐event analysis (a Cox proportional hazards model, package survival, Therneau, 2013) was used to compare the timing of dispersal among strains. Because we were also interested in possible changes in the timing of dispersal due to accidental selection in the cultures, we included the time that a strain had been in culture as a factor (see Supporting Information Table S1), as well as its interaction with the factor strain. Contrasts among treatments were assessed with the least‐squares means method of the package lsmeans with a Tukey adjustment of the probabilities (Lenth, 2016). We further investigated the effect of time in culture on the timing of dispersal per strain using a similar Cox proportional hazards model. These same data were used to calculate the proportion of predators that had dispersed during the interaction, defined as the total number of dispersed predators divided by the sum of the total number of dispersers plus the predators on the experimental leaf, yielding one proportion of dispersed predators per strain per replicate.

The interaction period was taken as the time interval between predator introduction and prey elimination, that is, the first day without prey on the experimental leaf. A similar time‐to‐event analysis was used as above, with time to prey elimination as dependent variable and strain and time in culture as factors.

The total number of predators was taken as the cumulative number of predators that dispersed from the leaf until the end of the experiment, when all predators had dispersed from the experimental patch. Differences in the total number of predators among strains, the effect of the time in culture, as well as their interaction were assessed with a GLM with quasi‐Poisson error distribution. Contrasts among treatments were assessed as explained above.

Furthermore, we tested for correlations among the proportion of predators that dispersed during the interaction, the interaction period, and the total number of predators with GLMs with a Poisson error distribution for the interaction period and a quasi‐Poisson error distribution for the total numbers of predators. In these analyses, strain and time in culture were used as fixed factors. All statistical analyses were performed with R version 3.0.1 (R Development Core Team 2017).

## 3 RESULTS

### 3.1 Sequencing of the COI and ITS genes

The sequences of both COI and ITS genes showed that the mites belonged to the species *P. persimilis*. All strains, including the Koppert line, had a COI sequence identical to the KF966638 entry in GenBank and an ITS sequence identical to the HQ404818 entry (Tsolakis, Tixier, Kreiter, & Ragusa, 2012).

### 3.2 Dispersal experiments

**Figure 3:**
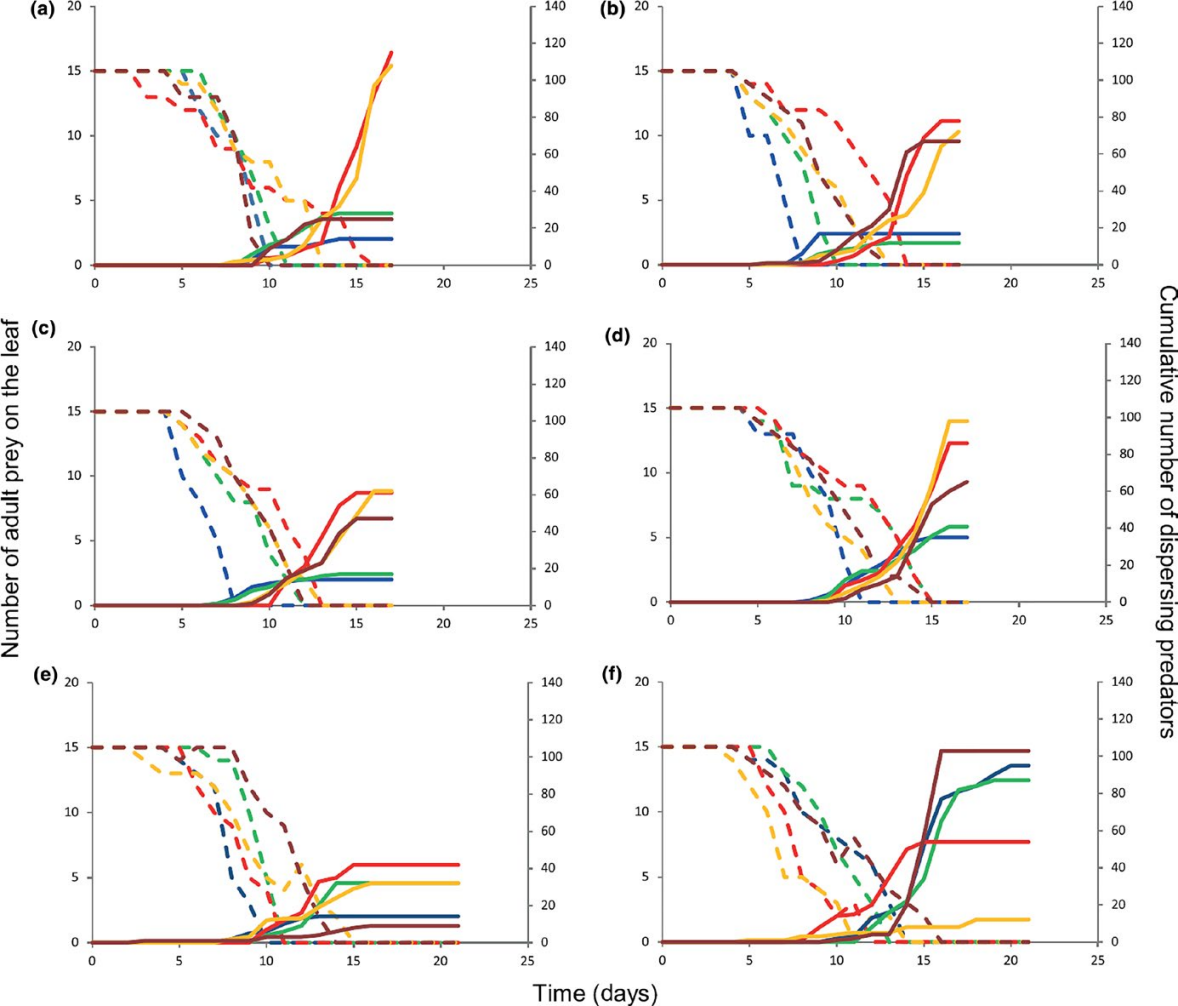
Predator– prey (*P. persimilis–T. urticae*) population dynamics in the wind tunnel experiments. Shown are the adult prey on the leaf (dashed lines, left‐hand axis) and cumulative number of dispersing predators (solid lines, right‐hand axis). The presented strains demonstrate the variation in prey exploitation and cumulative number of dispersers in every replicate. (a) Samandag, (b) Koyunoglu, (c) Kusalanı, (d) Uzunbag, (e) Trabia, and (f): Alcamo. Blue: Replicate 1, green: Replicate 2, red: Replicate 3, orange: Replicate 4, and purple: Replicate 5. For the population dynamics of all strains, see Supporting Information Figures S1 and S2

There was variation in the dynamics of the prey and predators among replicates and strains (Figure 3, Supporting Information Figures S1 and S2). In all cases, predators initiated dispersal while there were still prey present on the leaf.

**Figure 4:**
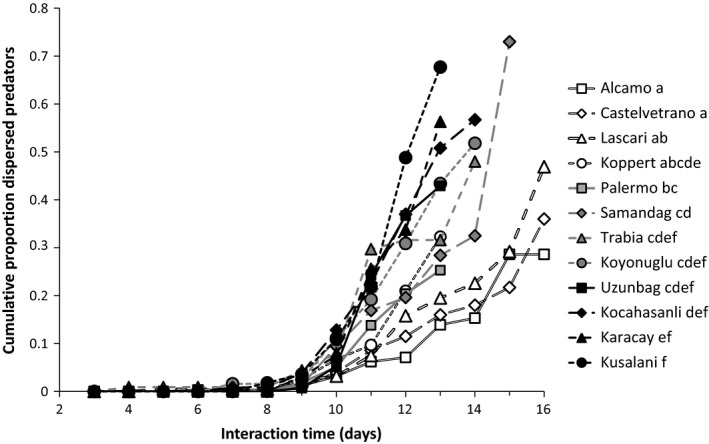
The cumulative proportion of predators that dispersed from experimental arenas during the interaction period. One adult female predator was released on an experimental arena consisting of a rose leaf infested with spider‐mite prey. Shown is the cumulative aerial dispersal of predators (the female and her offspring) from this patch. Curves represent the dispersal per strain (Figure 1, five replicates per strain). White symbols indicate more Killer‐like strains, black symbols more Milker‐like strains. Letters behind the strain names indicate significant differences among strains (contrasts a Cox mixed effects proportional hazards model)

There was a significant interaction between timing of dispersal and the time that strains had been in culture (Cox mixed effects proportional hazards: *χ* ^2^ = 63.7, *df* = 11, *p* < 0.001). The timing of dispersal varied significantly among strains (Figure 4). The strains from Alcamo, Castelvetrano, Lascari, and Koppert showed a more Killer‐like dispersal pattern (i.e., dispersal toward the end of the interaction period), whereas those from Uzunbag, Kocahasanli Karacay, and Kusalani were more Milker‐like (Figure 4). The strains from Karacay, Kocahasanli, Samandag, and Uzunbag showed a significant effect of time in culture on the timing of dispersal (likelihood ratio = 17.0, *df* = 1, *p* < 0.001; *L*‐ratio = 9.8 *df* = 1, *p* = 0.0017; *L*‐ratio = 21.3, *df* = 1, *p* < 0.001; *L*‐ratio = 12.7, *df* = 1, *p* = 0.0004, respectively). Except for the strain from Karacay, these strains showed a trend toward dispersing later and less with increasing time in culture, suggesting that there was some selection toward more Killer‐like behavior (Supporting Information Figure S3). However, none of the other strains showed such a significant effect.

**Figure 5:**
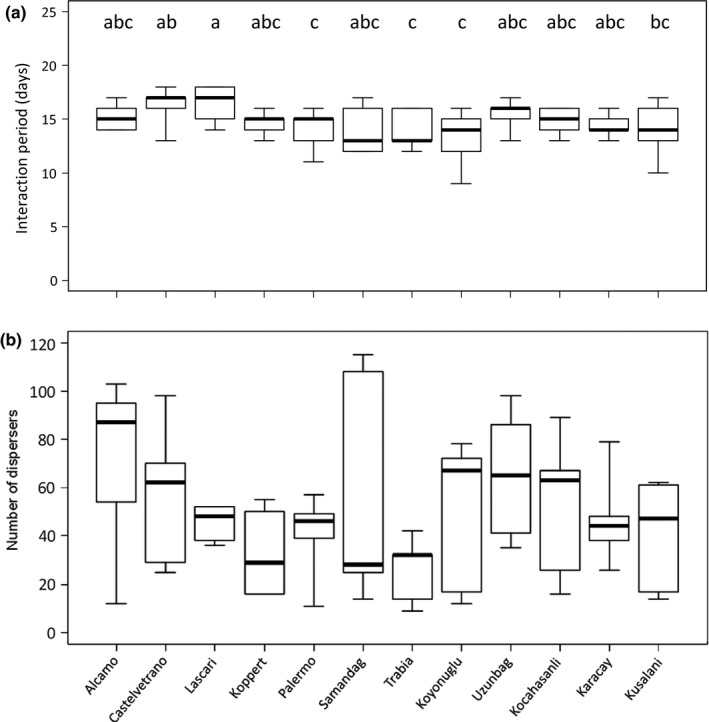
The median interaction period (a) and the median total numbers of predators (b) produced during the interaction by the strains described in Figure 1. Vertical thick lines show medians, boxes show 25th and 75th percentiles and whiskers give minima and maxima. Letters above the boxes in (a) indicate significance of differences among strains (contrasts with the package lsmeans with a Tukey adjustment, Length 2016 after a Cox mixed effects proportional hazards model). For clarity reasons, we present the median interaction time per strain in (a) instead of a graph of the cumulative number of populations that went extinct. However, the analysis of the interaction period was carried out with a time‐to‐event analysis

The interaction period, that is, the time between predator introduction and prey elimination, varied significantly with the interaction between strain and time in culture (Figure 5a, Cox mixed effects proportional hazards: *L*‐ratio = 22.1, *df* = 11, *p* = 0.024). The interaction period increased significantly with time in culture for the strain from Trabia (*L*‐ratio = 5.19, *df* = 1, *p* = 0.023); no such effect was found for the other strains.

The total number of dispersing predators did not differ significantly among strains (Figure 5b, GLM: *F*_11,47_ = 1.07, *p* = 0.41), and there was no significant effect of time in culture on the total numbers of dispersers (*F*_1,58_ = 3.24, *p* = 0.078). Of all collected strains, that from Alcamo produced the highest median number of dispersers and that from Samandag the lowest (Figure 5b).

**Figure 6:**
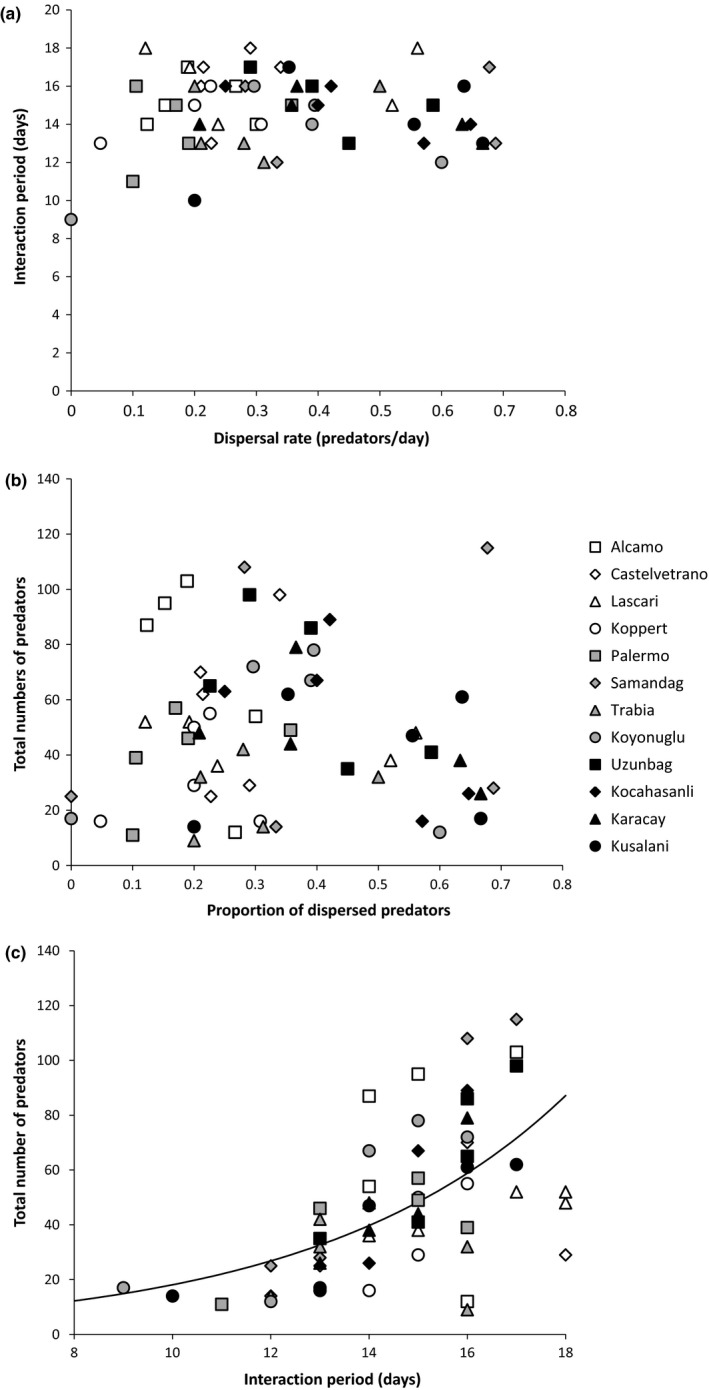
The relation between the proportion of predators dispersing during the interaction and the interaction period (a) or the total numbers of predators produced on a patch (b) and the relation between the interaction period and the total numbers of predators (c). Different symbols indicate the different strains as explained in the legend. The curve in (c) is fitted with a GLM

According to the Milker–Killer theory, the interaction period and the total numbers of predators at the end of the interaction period should be positively related to the dispersal rate during the interaction. We found little evidence for this (Figure 6a, dispersal vs. interaction period: GLM: χ^2^ = 0.085, *df* = 1, *p* = 0.77; Figure 6b, dispersal vs. total number of predators: *F*_1,58_ = 0.005, *p* = 0.94). The interaction period also did not differ significantly among strains (interaction period: GLM: *χ* ^2^ = 4.15, *df* = 11, *p* = 0.965; total number of predators: *F*_11,47_ = 1.07, *p* = 0.41). Nevertheless, there was a significant positive correlation between the interaction period and the total number of predators (Figure 6c, GLM: *F*_1,58_ = 47.15, *p* < 0.001).

## 4 DISCUSSION

Earlier work showed first evidence for the existence of variation in exploitation strategies by the predatory mite *P. persimilis*, one isofemale line resembling the Killer strategy in which predators started dispersing at prey depletion, the other isofemale line resembling the Milker strategy, in which predators started dispersing before prey elimination, thus leaving food for their offspring (Pels & Sabelis, 1999). Here, we investigated whether the Killer and the Milker strategies occur in nature, using recently collected strains from various local populations. We used the dispersal rate during the predator–prey interaction as a criterion to determine which exploitation strategy the predators employ. Our results showed significant variation in dispersal rates across strains (Figure 4). The strains Castelvetrano, Alcamo, Lascari, and Koppert had lower dispersal rates during the interaction period, and we conclude that they employed a more Killer‐like dispersal strategy. In contrast, the strains Uzunbag, Kocahasanli, Karacay, and Kusalani employed a more Milker‐like dispersal strategy. Given the differences in the dispersal rates, we expected to observe the predicted consequences for the predator–prey dynamics and the cumulative number of dispersers; however, this was not the case. Higher dispersal rates did not result in a prolonged interaction period and a higher production of dispersers (Figure 6); thus, we conclude that none of the strains belonged to the extreme Killer or Milker strategy. However, we did find a significant correlation between the interaction period and the total number of predators, which is in agreement with the prediction that a longer interaction period will result in a higher number of predators (Figure 6c). Perhaps, the predator strains with a longer interaction period had lower predation rates combined with high conversion rates of consumed prey into eggs. This would also result in a prolonged growth of the prey populations and, consequently, more food for the predators’ offspring.

The local population dynamics may of course vary with prey characteristics such as prey dispersal and antipredation strategies. If the prey mites respond flexibly to predation pressure, the predator exploitation strategy might not have much net effect on the interaction period and the cumulative number of predator dispersers. In predator–prey interactions, it is to be expected that prey disperse as well, either to avoid predation or to find a better host plant. Milker–Killer‐like strategies may occur in spider mites exploiting their host plant as well; spider mites also overexploit their food source, and Killer‐like predator exploitation selects for higher prey dispersal (Sabelis, Van, Pels, Egas, & Janssen, 2002). Even though dispersing prey were never observed in the wind tunnels, we cannot exclude the possibility that prey dispersed from the arena to the trap, and the setup of the wind tunnels did not allow for observations on antipredator behavior. If the interaction period is also dependent on such condition‐dependent prey behavior, it is still an open question what would be the best exploitation strategy for the predators.

We tried to maintain the natural variation in dispersal behavior of the predators in the laboratory using closed rearing units, which allowed the predators to leave the prey patch, but to which they could subsequently return. The mites were collected from the field and subsequent adaptation to the rearing conditions might have affected their dispersal behavior (Pettit, Greenlees, & Shine, 2016). Indeed, we found for some strains that the dispersal tendency significantly decreased with a longer time in the laboratory (Supporting Information Figure S3). However, the majority of the strains showed no such significant tendency.

Genetic variation among predators within each strain may also contribute to variation among replicates. In contrast to Pels and Sabelis (1999), we did use isofemale lines but tested one family in each replicate (each experiment was started with one adult female predator). The experiments of Pels and Sabelis (1999) show much less variation in interaction time among replicates than the experiments reported here, suggesting that there was genetic variation for prey exploitation within the strains studied here. This variation suggests that there is a genetic component for dispersal tendency in the presence of prey (see also Jia, Margolies, Boyer, & Charlton, 2002), but the question remains to what extent this variation is heritable. To test this, selection should be performed for Milker and Killer lines of predators. Creating such lines would provide an important tool for further studies of the evolution and maintenance of variation in prey exploitation strategies and their effects on local and global population dynamics.

Dispersal is generally treated as a phenomenon that is either only genetically determined and linked to life‐history traits (e.g., Stevens et al., 2013, 2014), or plastic and dependent on context (e.g., Bitume, Bonte, Ronce, Olivieri, & Nieberding, 2014; Bitume et al., 2013; Clobert, Le Galliard, Cote, Meylan, & Massot, 2009). However, for a comprehensive understanding of dispersal, these perspectives should be united (Bonte & Dahirel, 2017). In the current study, we did not observe the population dynamical consequences predicted by the model of van Baalen and Sabelis (1995). We suggest that the discrepancy is due to the model assumption of a fixed dispersal probability during the interaction period. In our experiment, dispersal was probably affected by predator density and food availability. All predators from all strains and in all replicates started dispersing close to, but still before prey elimination. It remains to be investigated how the condition of the individual and its environment affects its dispersal behavior.

## CONFLICT OF INTEREST

The authors declare no conflict of interest.

## AUTHOR CONTRIBUTION

MWS and AMR designed the experiments; AMR sampled the mites used for this study, performed the experiments, and wrote the main manuscript; AMR and AJ prepared figures; all authors contributed to the conception of the ideas, and all authors edited and reviewed the manuscript.

## ETHICAL APPROVAL

All applicable institutional and/or national guidelines for the care and use of animals were followed.

## DATA AVAILABILITY

Data are available in Dryad, https://doi.org/10.5061/dryad.8hs46jv.
