# Supplementary figures and images for: Prey exploitation and dispersal strategies vary among natural populations of a predatory mite
Source: Ecol Evol. 2018 Oct 13;8(21):10384–94. doi: 10.1002/ece3.4446 (PMC6238141; doi:10.1002/ece3.4446)

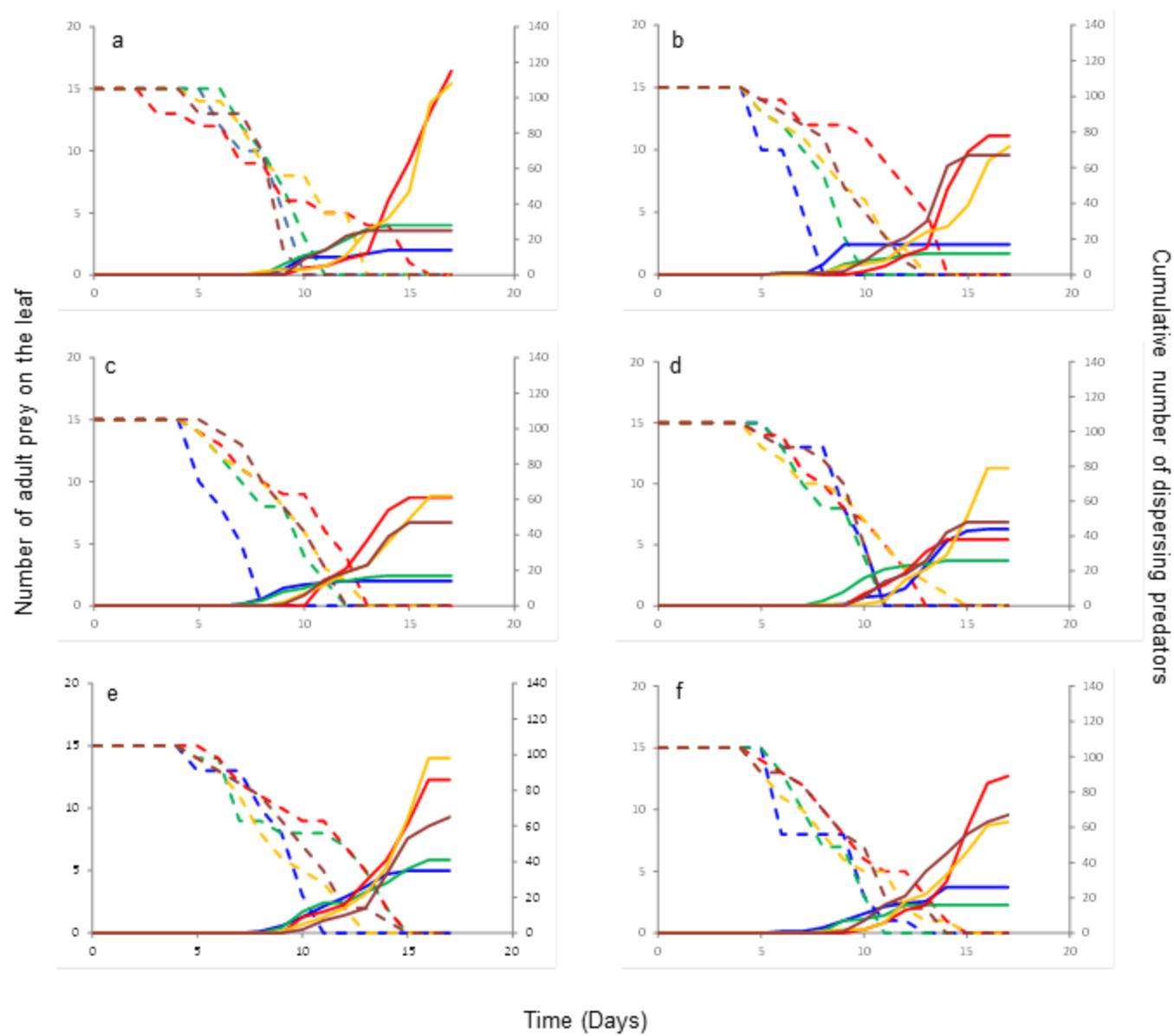

Supplement: Supplementary file 1 [file ECE3-8-10384-s001.pdf]

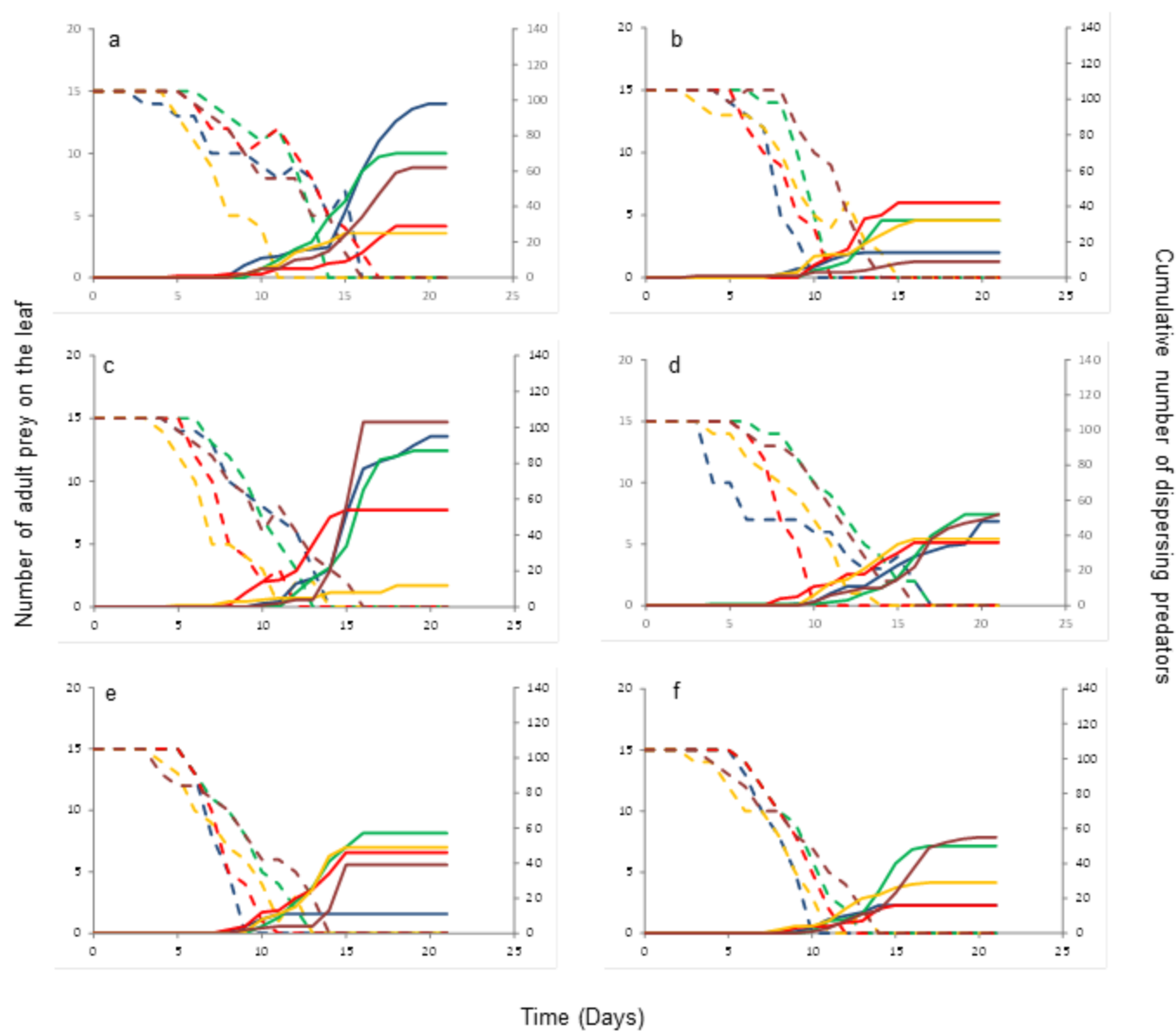

Supplement: Supplementary file 2 [file ECE3-8-10384-s002.pdf]

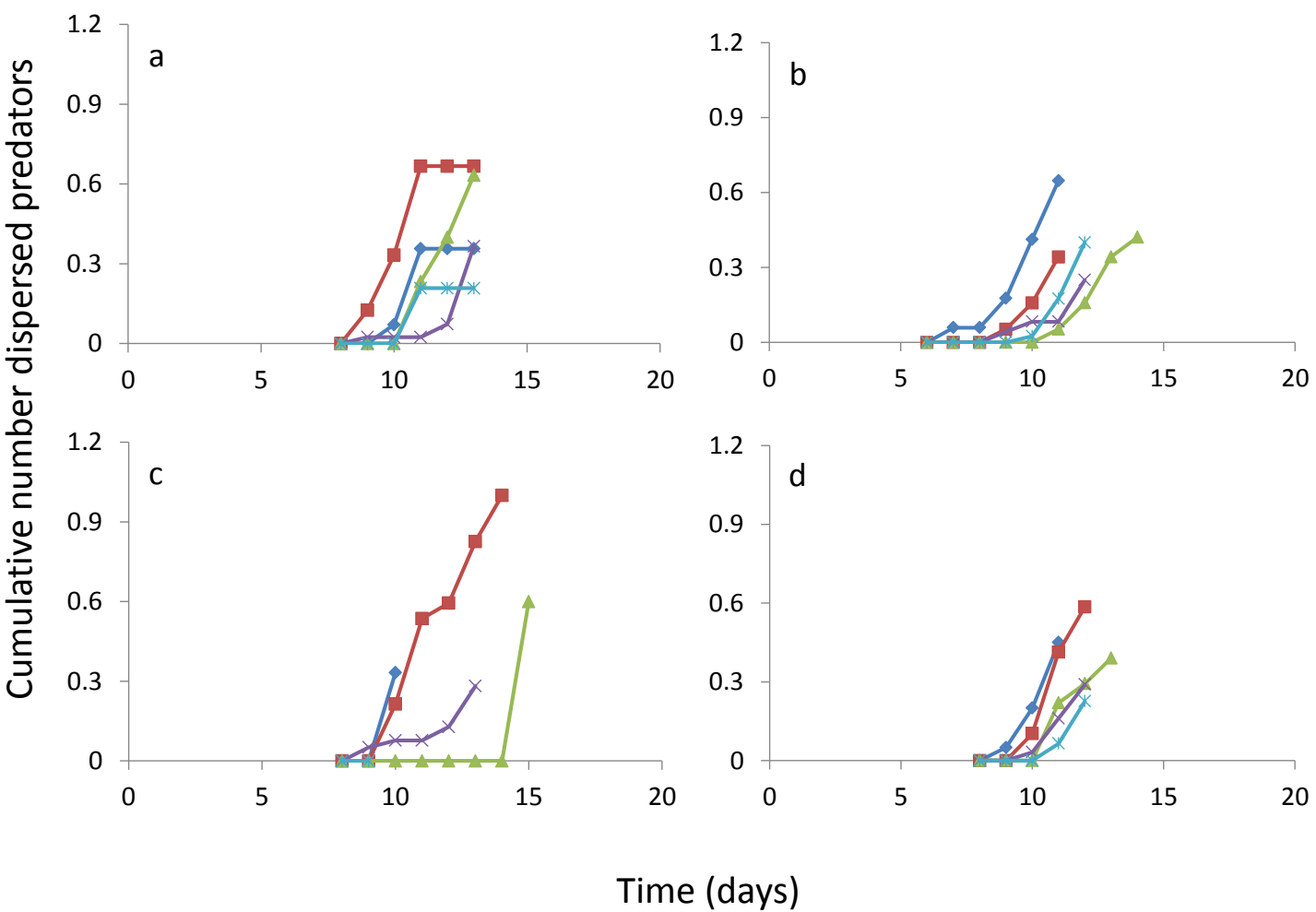

Supplement: Supplementary file 3 [file ECE3-8-10384-s003.pdf]
