# Supplementary material for: Prey exploitation and dispersal strategies vary among natural populations of a predatory mite
Source: Ecol Evol. 2018 Oct 13;8(21):10384–94. doi: 10.1002/ece3.4446 (PMC6238141; doi:10.1002/ece3.4446)
Supplement: Supplementary file 4 [file ECE3-8-10384-s004.docx]

**Prey exploitation and dispersal strategies vary among natural populations of a predatory mite**

A.M. Revynthi^a^, M. Egas*^a^, A. Janssen^a^ and M.W. Sabelis^a,†^

^a^ Institute of Biodiversity and Ecosystem Dynamics, University of Amsterdam, P.O. box 94248, 1090 GE, Amsterdam, The Netherlands

^†^deceased 7 January, 2015

* Corresponding author: [egas@uva.nl](mailto:c.j.m.egas@uva.nl)

A. M. Revynthi: [A.M.Revynthi@uva.nl](mailto:A.M.Revynthi@uva.nl)

A. Janssen: [Arne.Janssen@uva.nl](mailto:Arne.Janssen@uva.nl)

**Supplementary material**

| Country | Strain | Date of collection | Date of culture | Replicate | Date of testing |
| --- | --- | --- | --- | --- | --- |
| Turkey | Samandağ | 17-Jul-2013 | 22-Jul-2013 | a | 25-Nov-2013 |
|  |  |  |  | b | 23-Dec-2013 |
|  |  |  |  | c | 13-Jan-2014 |
|  |  |  |  | d | 10-Feb-2014 |
|  |  |  |  | e | 3-Mar-2014 |
|  | Koyunoğlu | 16-Jul-2013 | 22-Jul-2013 | a | 25-Nov-2013 |
|  |  |  |  | b | 23-Dec-2013 |
|  |  |  |  | c | 13-Jan-2014 |
|  |  |  |  | d | 10-Feb-2014 |
|  |  |  |  | e | 3-Mar-2014 |
|  | Kuşalanı | 16-Jul-2013 | 22-Jul-2013 | a | 25-Nov-2013 |
|  |  |  |  | b | 23-Dec-2013 |
|  |  |  |  | c | 13-Jan-2014 |
|  |  |  |  | d | 10-Feb-2014 |
|  |  |  |  | e | 3-Mar-2014 |
|  | Karaçay | 17-Jul-2013 | 22-Jul-2013 | a | 25-Nov-2013 |
|  |  |  |  | b | 23-Dec-2013 |
|  |  |  |  | c | 13-Jan-2014 |
|  |  |  |  | d | 10-Feb-2014 |
|  |  |  |  | e | 3-Mar-2014 |
|  | Uzunbağ | 16-Jul-2013 | 22-Jul-2013 | a | 25-Nov-2013 |
|  |  |  |  | b | 23-Dec-2013 |
|  |  |  |  | c | 13-Jan-2014 |
|  |  |  |  | d | 10-Feb-2014 |
|  |  |  |  | e | 3-Mar-2014 |
|  | Kocahasanlı | 19-Jul-2013 | 22-Jul-2013 | a | 25-Nov-2013 |
|  |  |  |  | b | 23-Dec-2013 |
|  |  |  |  | c | 13-Jan-2014 |
|  |  |  |  | d | 10-Feb-2014 |
|  |  |  |  | e | 3-Mar-2014 |
| Sicily | Castelvetrano | 4-Jun-2014 | 8-Jun-2014 | a | 22-Sep-2014 |
|  |  |  |  | b | 4-Nov-2014 |
|  |  |  |  | c | 1-Dec-2014 |
|  |  |  |  | d | 26-Jan-2015 |
|  |  |  |  | e | 17-Feb-2015 |
|  | Trabia | 5-Jun-2014 | 8-Jun-2014 | a | 22-Sep-2014 |
|  |  |  |  | b | 4-Nov-2014 |
|  |  |  |  | c | 1-Dec-2014 |
|  |  |  |  | d | 26-Jan-2015 |
|  |  |  |  | e | 17-Feb-2015 |
|  | Alcamo | 4-Jun-2014 | 8-Jun-2014 | a | 22-Sep-2014 |
|  |  |  |  | b | 4-Nov-2014 |
|  |  |  |  | c | 1-Dec-2014 |
|  |  |  |  | d | 26-Jan-2015 |
|  |  |  |  | e | 17-Feb-2015 |
|  | Lascari | 5-Jun-2014 | 8-Jun-2014 | a | 22-Sep-2014 |
|  |  |  |  | b | 4-Nov-2014 |
|  |  |  |  | c | 1-Dec-2014 |
|  |  |  |  | d | 26-Jan-2015 |
|  |  |  |  | e | 17-Feb-2015 |
|  | Palermo | 4-Jun-2014 | 8-Jun-2014 | a | 22-Sep-2014 |
|  |  |  |  | b | 4-Nov-2014 |
|  |  |  |  | c | 1-Dec-2014 |
|  |  |  |  | d | 26-Jan-2015 |
|  |  |  |  | e | 17-Feb-2015 |
| Koppert | Koppert | 1-Jan-1994 | 4-Sep-2014 | a | 22-Sep-2014 |
|  |  |  |  | b | 4-Nov-2014 |
|  |  |  |  | c | 1-Dec-2014 |
|  |  |  |  | d | 26-Jan-2015 |
|  |  |  |  | e | 17-Feb-2015 |

**Table S1.** Timetable of collection, starting laboratory culture, and testing each strain of *P. persimilis*.

**Fig. S1.** Population dynamics of adult prey (*T. urticae*) on the leaf (dashed lines, left-hand vertical axis) and cumulative number of dispersing predators (*P. persimilis*) (solid lines, right-hand vertical axis). Different color corresponds to different replicate. Blue: replicate 1, Green: replicate 2, Red: replicate 3, Orange: replicate 4 and Purple: replicate 5. a: Samandag, b: Koyunoglu, c: Kusalanı, d: Karacay, e: Uzunbag, f: Kocahasanli.

**Fig. S2**. Population dynamics of adult prey (*T. urticae*) on the leaf (dashed lines, left-hand vertical axis) and cumulative number of dispersing predators (*P. persimilis*) (solid lines, right-hand vertical axis). Different color corresponds to different replicate. Blue: replicate 1, Green: replicate 2, Red: replicate 3, Orange: replicate 4 and Purple: replicate 5. a: Castelvetrano, b: Trabia, c: Alcamo, d: Lascari, e: Palermo, f: Koppert.

**Fig. S3.** Effect of time in culture on the timing of dispersal of the strains from a) Karacay, b) Kocahasanli, c) Samandag and d) Uzunbag. Except for the strain from a) Karacay, these strains showed a trend towards dispersing later and less with increasing time in culture, suggesting that there was some selection towards more Killer-like behavior. Different color corresponds to different time in culture. Dark blue: 18 weeks in culture, Red: 22 weeks, Green: 25 weeks, Purple: 29 weeks and Light blue: 32 weeks.
